# Navigating miscarriage in Jordan: understanding emotional responses and coping strategies

**DOI:** 10.1186/s12884-023-06075-6

**Published:** 2023-10-26

**Authors:** Esra’ Taybeh, Shereen Hamadneh, Zina Al-Alami, Rana Abu-Huwaij

**Affiliations:** 1 Department of Applied Pharmaceutical Sciences and Clinical Pharmacy, Faculty of Pharmacy, Isra University, Amman, Jordan; 2 Department of Maternal and Child Health, Princess Salma Faculty of Nursing, Al al-Bayt University, Mafraq, Jordan; 3 Department of Basic Medical Sciences, Faculty of Allied Medical Sciences, Al-Ahliyya Amman University, Amman, Jordan; 4 Department of Pharmacy, College of Pharmacy, Amman Arab University, Amman, Jordan

**Keywords:** Miscarriage, Emotion, Social support, Jordan

## Abstract

**Background:**

Miscarriages account for 20% of clinically confirmed pregnancies and up to 50% of all pregnancies and is considered one of the most heartbreaking events experienced by women. The current study aimed to explore participants’ perceptions and practices and how they link with the negative emotions of miscarriage.

**Methods:**

In this cross-sectional study a web-based questionnaire was used to gather data from 355 women living in Jordan who had experienced a previous miscarriage. The questionnaire consisted of four sections, including socio-demographic information, experience with miscarriage, emotions after the experience, and self-care practices. Participants were recruited through social media platforms from April to August 2022. Data were analyzed using SPSS, and descriptive statistics, chi-square test, and binomial regression were performed to examine the results.

**Results:**

The results show that the majority of participants were in the age group of 22–34 years and a larger percentage of participants hold a Bachelor’s degree and were employed. All participants had experienced a previous miscarriage with 53.8% having one, 27.0% having two, and 19.2% having three or more miscarriages. In addition, most miscarriages did not have an explanation for their cause (77.5%), but vaginal bleeding was the most reported symptom (55.2%) and surgical management was predominant (48.7%). Most participants reported adequate emotional support from partners and family (63.7% and 62.3%, respectively). Almost half (48.7%) of the respondents felt like they had lost a child and those who did not receive any social support had a higher association with the same feeling (p = 0.005). Of the participating women, 40.3% decided to postpone another pregnancy while 20.0% planned for a subsequent pregnancy. The feeling of shame regarding the miscarriage was the main driver for women to get pregnant again (Odd ration [OR] 2.98; 95% confidence interval (CI) 1.31–6.82; *p* = 0.01).

**Conclusions:**

The findings highlight the emotional impact of miscarriage on women and the need for proper support and self-care practices.

**Supplementary Information:**

The online version contains supplementary material available at 10.1186/s12884-023-06075-6.

## Introduction

Pregnancy can be experienced as a joyful event by many, but miscarriage is a stressful experience for women and their families. Despite the major advances in medicine and obstetrics, women are still facing pregnancy loss, stillbirth, and neonatal deaths [1]. An estimated 23 million miscarriages occur yearly, translating to 44 pregnancy losses each minute worldwide [2].

Miscarriage costs have an impact on people, healthcare systems, and society. It was thought to damage the UK’s economy with expenses reaching £471 million annually [2]. In addition, a high percentage of maternal mortality is frequently linked to miscarriage. Besides cost and loss of life, miscarriages may affect patients’ psychological and emotional responses [3–5]. At the time of miscarriage, many women experience a period of intense emotional distress that increases the risk of anxiety, depression, and suicide [6]. A prospective, multicenter cohort study of 537 women following a miscarriage found that 18% of women had post-traumatic stress, 17% had moderate or severe anxiety, and 6% had moderate or severe depression [7]. Also, mothers in Kenya were often blamed following a miscarriage, so they would consequently not seek care after one unless there was a complication [8]. Miscarriage consequences tend to improve after six weeks to several months, but some women may continue to experience negative symptoms for a longer time [6]. In the specific context of Jordan, cultural factors play a crucial role in shaping women’s experiences following a miscarriage. One important aspect to consider is that pregnancy outside of marriage is not culturally accepted in Jordan. This cultural norm has significant implications for women who experience a miscarriage outside of the institution of marriage, as it may affect their social support networks, access to resources, and overall experiences during this challenging time. Furthermore, it is important to note that the percentage of Arab individuals seeking psychological help is typically lower compared to Western countries [9]. As a result, the majority of studies examining the psychological impact of miscarriage have been conducted in Western countries [10].

During the miscarriage period, various coping strategies, including self-care practices, exist to handle the emotions of women. Self-care refers to intentional actions taken to promote physical, mental, and emotional well-being [11]. These strategies encompass searching for information, positively reappraising the situation, and seeking social support [12]. Research shows that societal norms impact a variety of aspects pertaining to the experience of miscarriage, but such aspects are rarely considered while understanding different context and perceptions of adverse pregnancy outcomes [13].

Since the body of work on miscarriage is scant, this paper presents a different context than what was previously discussed in the literature and shows not only the variations to the experience of miscarriage but also continuities. The study obtained the perceptions and practices of participants in Jordan and considered how they were linked with the management of difficult miscarriage-related emotions. The present work sheds new light on the experience of miscarriage in Jordan and it contributes to growing literature on women’s health in Middle Eastern countries which share similar sociocultural elements.

## Method

### Study instrument

Our questionnaire design involved incorporating a literature review and expert input. Through an extensive literature review, we identified relevant constructs and measurement items for our study. We sought the expertise of gynecologists to refine the questionnaire and ensure its relevance. Additionally, we reviewed and adapted previous web-based questionnaires [4, 14, 15], making necessary modifications to suit our research objectives. The questionnaire aimed to capture participants’ subjective reflections on emotional well-being post-miscarriage, explore coping strategies, and identify influential factors. Therefore, the questionnaire consisted of four sections. Section one contained the socio-demographic information of the participants. Section two comprised three checkbox questions on the experience of miscarriage. The questions addressed the symptoms, the medical causes, and the treatment of miscarriage. Section three comprised six questions focusing on the emotions of women after experiencing a miscarriage covering a scale of “strongly disagree” to “strongly agree” as well as the strategies used to control negative emotions including social support after miscarriage. Section four focused on questions with multiple choice answers, without the ability to provide additional information or select an “other” category, on the practices and behaviors of women in terms of planning or postponing an upcoming pregnancy. The questionnaire was initially designed in English and a back-translation was performed by native Arabic speakers. Before launching, the survey was piloted with 20 participants to assess the content readability, examine question flow, and test the functionality of the survey link. Minor modifications such as rephrasing a question were considered. The responses from the pilot testing were not included in the final analysis. The Cronbach’s Alpha reliability analysis test to measure the internal consistency of the questionnaire was performed and was observed to be 0.708.

### Sampling and data collection

A convenience sample of women who experienced a previous miscarriage was recruited for this study. The estimated sample size of 378 was calculated based on a prevalence rate of 20% for miscarriages, a 95% confidence interval, and a 5% margin of error. However, due to recruitment challenges and potential participant dropout, the final collected sample size was 355. Despite this slight deviation, the collected data still provides valuable insights and contributes to our research objectives.

The survey was performed online and anonymously through the Google Forms platform and the participants were approached through social media applications (i.e., Facebook and WhatsApp). The study was approved by the Ethical Approval Committee of the Institutional Review Board of Al-Ahliyya Amman University (approval number: MM 1/ 4-2022).

The survey was open for participants living in Jordan from April 2022 to August 2022. The first page of the survey displayed the study aims, the anonymous collection of data, and the right to discontinue participating at any time. Two screening questions were used to ensure that participants were married (pregnancy outside of marriage is not culturally accepted in Jordan) and had a previous experience of miscarriage. Before starting to fill the questionnaire, informed consent was obtained from participants by a check in the “Agree” box.

Data collection was started by sending an invitation text to their network via WhatsApp with the survey link. The invitation was also posted on several Facebook groups. A snowball sampling technique was used where referrals from participants to invite their family, friends, and colleagues to participate could be done by forwarding the online survey link. There were no incentives or financial rewards offered to participants.

### Data analysis

All data were analyzed using SPSS version 26 (SPSS Inc., USA). Descriptive statistical analysis (i.e., frequencies and percentages) was performed to present the socio-demographic characteristics and the emotional responses after experiencing miscarriage. Chi-square test was used to test for significant association between selected categorical variables, such as the received social support and participant emotions. Binomial regression was performed to examine the predictive effect of different emotions (the categorical independent variables) on the timing of an upcoming pregnancy. Results with a p-value < 0.05 were considered statistically significant.

## Results

### Basic characteristics of participants

A total of 355 respondents agreed to participate and completed the survey. The characteristics of the participants are shown in Table 1. The largest percentage of participants (47.0%) according to age was the group of 22–34 years and 50.7% of the participants live in North Jordan. The rate of female respondents who hold a Bachelor’s degree (41.1%) was significantly higher than that of other degrees. Even with a high employment proportion (58.9%), approximately half of the participants (46.8%) had a monthly income < 500 JD. All participants experienced a previous miscarriage with 53.8% having one, 27.0% having two, and 19.2% having three or more miscarriages. Of the participants, 11.5% have no children while 42.8% have 2–3 children in total.

**Table 1:** Characteristics of Participants (n = 355)

Character		Frequency (%)
Age	< 22 years	7 (2.0)
	22–34 years	167 (47.0)
	35–44 years	139 (39.2)
	> 44 years	42 (11.8)
Residency	North Jordan	180 (50.7)
	Central Jordan	151 (42.5)
	South Jordan	24 (6.8)
Educational level	Secondary education or less	91 (25.6)
	College degree	68 (19.2)
	Bachelor’s degree	146 (41.1)
	Postgraduate degree	50 (14.1)
Employment	Employed	209 (58.9)
	Unemployed	146 (41.1)
Family income	< 500 JD	166 (46.8)
	500–999 JD	110 (31.0)
	1,000–1,500 JD	48 (13.5)
	> 1,500 JD	31 (8.7)
Number of children	None	41 (11.5)
	One child	55 (15.5)
	2–3 children	152 (42.8)
	4 or more	107 (30.1)
Number of miscarriages	Once	191 (53.8)
	Twice	96 (27.0)
	Three or more	68 (19.2)

### Symptoms, medical causes, and treatment of miscarriage

Table 2 shows the medical characteristics of the miscarriage among participants. The most reported symptom of miscarriage was vaginal bleeding (55.2%) followed by abdominal pain (45.1%). The known causes of the miscarriage were reported by some women and included antiphospholipid syndrome (6.8%) and abnormal chromosomes (6.5%). However, around three-quarters (77.5%) reported that they were not given a cause for the miscarriage. The treatment options that were reported by participants included surgical interventions (48.7%), expectant management (36.1%), and medication (15.2%). Cross-tabulation found that women who live in South Jordan were more likely to be medically managed and less likely to perform curettage surgery (p < 0.001). Moreover, the results showed that those with three or more miscarriages were more likely to be managed via surgical evacuation (p = 0.03).

**Table 2:** Medical Characteristics of the Miscarriage

Experience		Frequency (%)
Experienced symptoms*	Vaginal bleeding	196 (55.2%)
	Abdominal pain	160 (45.1%)
	Passing tissues or clots	93 (26.2%)
	Reduced pregnancy signs (i.e., nausea and vomiting, breast tenderness, tiredness)	60 (16.9%)
	No symptoms (suddenly discovered)	75 (21.1%)
Causes of miscarriage	Chromosomal abnormality	23 (6.5%)
	Uterine factor	12 (3.4%)
	Antiphospholipid syndrome	24 (6.8%)
	Endocrine abnormality	4 (1.1%)
	Infections	17 (4.8%)
	Unexplained	275 (77.5%)
Treatment of miscarriage	Expectant management	128 (36.1%)
	Medical management	54 (15.2%)
	Surgical management	173 (48.7%)

* Multiple answers were allowed

### Emotional effects of and healing after miscarriage

**Fig. 1:**
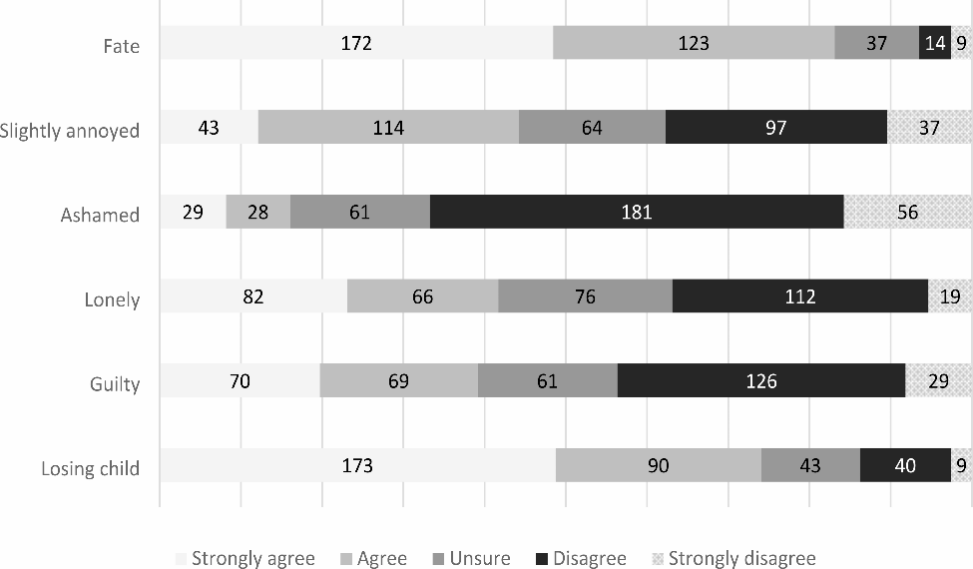
Emotional response after experiencing miscarriage (n = 355)

Figure 1 shows the results of our study that surveyed participants about their emotions and thoughts after experiencing a miscarriage. Nearly half (48.7%) of the respondents felt like they had lost a child while 23.1% felt lonely. A smaller percentage of participants felt guilty (19.7%) or slightly upset (12.1%). On the other hand, 48.5% of the participants believed that the miscarriage was their fate and were able to accept it.

**Fig. 2:**
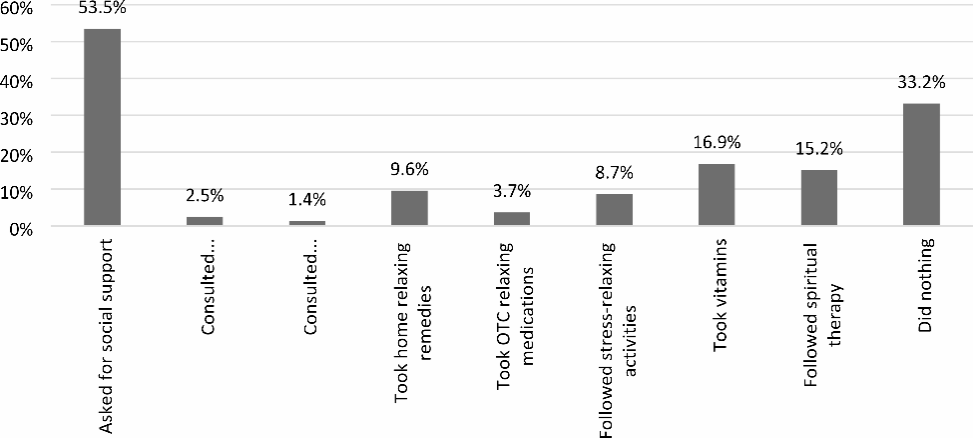
Strategies used to control negative emotions after miscarriage, where multiple answers were allowed

Figure 2 highlights the strategies that helped participants reduce their negative emotions after a miscarriage. The most reported approach was seeking social support, with 53.5% of participants reporting that this helped. The use of vitamins (16.9%) and spiritual rituals (15.2%) were also reported as helpful. However, it is important to note that 33.2% reported doing nothing to alleviate their negative emotions.

**Fig. 3:**
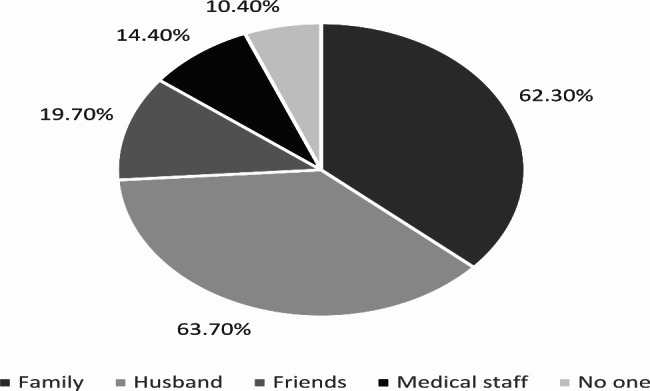
Sources of emotional support received by participants after experiencing a miscarriage

Figure 3 shows the sources of emotional support received by participants after experiencing a miscarriage. Most of the participants (63.7%) reported receiving adequate emotional support from their partners, and a similar proportion (62.3%) received support from their family. Friends were a source of support for 19.7% of the participants, and 14.44% reported receiving adequate emotional support from medical staff. On the other hand, 10.4% of participants reported not receiving any social support.

The data collected through the survey questions allowed us to identify significant associations between feelings and social support. The results showed that those who did not receive any social support were more likely to feel like they had lost a child (p = 0.005). However, other emotions such as feeling guilty, lonely, ashamed, annoyed, or accepting one’s fate were not associated with the presence or absence of social support (Table 3). It is important to note that the findings reflect associations rather than cause and effect.

**Table 3:** Sources of Emotional Support after Experiencing a Miscarriage and Their Effect on Participants’ Emotions (N, %)

Feelings	Received social support				No social support	*P*-value
	Family	Husband	Friends	Medical staff		
**Like child loss**	173 48.7%	173 48.7%	60 16.9%	44 12.4%	19 5.4%	0.005*
**Guilty**	88 24.8%	87 24.5%	41 11.5%	28 7.9%	17 4.8%	0.229
**Lonely**	101 28.5%	91 25.6%	43 12.1%	28 7.9%	15 4.2%	0.883
**Ashamed**	32 9.0%	39 11.0%	15 4.2%	9 2.5%	7 2.0%	0.503
**Annoyed**	90 25.4%	104 29.3%	26 7.3%	19 5.4%	18 5.1%	0.366
**Fate acceptance**	182 51.3%	193 54.4%	56 15.8%	38 10.7%	30 8.5%	0.664

*p-value was based on chi-square test and < 0.05 was considered significant

### Self-care practices after miscarriage

Of the participating women, only 20.0% planned for subsequent pregnancy and 40.3% decided to postpone pregnancy while 39.7% did not know whether they wished to become pregnant again. The feelings of child loss, guilt, or loneliness were initially associated with the decision of subsequent pregnancy, but after performing logistic regression, they were no longer significant (Table 4). It is the feeling of shame that was the driver for many women to get pregnant again. That is, women who felt ashamed of the miscarriage were three times more likely to get pregnant soon after (Odd ration [OR] 2.98; 95% confidence interval (CI) 1.31–6.82; *P* = 0.01).

Those who planned to get pregnant again focused on achieving a steadily progressing and healthy pregnancy. A percentage of 74.6% of them persevered with praying, 32.4% consumed fertility-enhancing herbs, 23.9% did cupping therapy, and 15.5% utilized medically assisted reproduction treatments. On the other hand, 45.5% of women who were not ready to get pregnant again used different birth control methods, such as intrauterine devices and oral contraceptives.

**Table 4:** Emotional Effect on Upcoming Pregnancy (N, %)

	Postponing pregnancy			Planned to be pregnant too early				*P*-value
	I waited more than two months before trying to become pregnant again	I used intrauterine birth control method immediately	I used other birth control method immediately	I had spiritual treatment/ praying/ Quran recitation	I had cupping therapy*	I used Vitex or Majorana plants	I had medical assisted conception	
**Like child loss**	81 22.8%	9 2.5%	31 8.7%	63 17.7%	15 4.2%	21 5.9%	11 3.1%	0.119
**Guilty**	35 9.9%	9 2.5%	17 4.8%	36 10.1%	12 3.4%	16 4.5%	3 0.8%	0.799
**Lonely**	44 12.4%	5 1.4%	17 4.8%	43 12.1%	12 3.4%	17 4.8%	7 2.0%	0.625
**Ashamed**	14 3.9%	2 0.6%	5 1.4%	18 5.1%	7 2.0%	11 3.1%	6 1.7%	0.010*

*p-value was based on binary logistic regression test and < 0.05 was considered significant

*The application of heated cups to create local suction on the skin

## Discussion

In this population-based study of women in Jordan who experienced miscarriage, multiple signs and symptoms were often reported. The most reported symptom among participants was vaginal bleeding (55.2%). A comparable percentage (53%) was reported by Sapra and colleagues (2016) among women who experienced miscarriage [16]. While vaginal bleeding is a sign of pregnancy loss [17], nausea and vomiting have been inversely associated with miscarriage. Our findings, consistently, showed that 16.9% of the respondents experienced reduced nausea and vomiting as an early sign of miscarriage. Understanding of the causes of pregnancy loss and the biologic mechanisms behind the spontaneous pregnancy termination is still very much unknown [16]. Expectedly in the present study, more than a quarter of the respondents did not know the cause of their miscarriage due to a lack of information. Building upon this observation, it is essential to highlight the need for further investigations aimed at identifying the causes of miscarriage and increasing knowledge in this field.

Results in the current study showed that the first choice for miscarriage management was surgical interventions (48.7%), followed by expectant management, and only a small percentage of women were treated by medications. In a review published in 2014, it was mentioned that expectant management, rather than surgical intervention, was the first-line approach [18]. Women usually are recommended to wait for two weeks for the miscarriage to complete. Nevertheless, if there are no signs of infection, it might be safe to continue with expectant management [19]. On the other hand, medical treatment based on our study was the least reported strategy. Although prostaglandin analogue (i.e., misoprostol) is a cost-effective choice compared to surgery and causes mild side effects [20–22], it is not widely accessible. It is recommended to investigate Jordanian physicians’ attitude toward surgery preferences and to evaluate the complication and effectiveness of management protocols for miscarriages in Jordan.

Regardless of the cause and treatment, miscarriage during early pregnancy is one of the most heartbreaking issues. It is reported that 20% of clinically confirmed pregnancies and up to 50% of all pregnancies are thought to experience miscarriage [23, 24]. Misconceptions and attitudes towards miscarriage vary in society, with some individuals viewing it as a normal and common occurrence, while others attach stigma and shame to it [13]. It was not surprising to note that around half (48.7%) of the respondents in our study felt like they had lost a child while others felt lonely, guilty, or upset. Miscarriage was previously reported to have a profound emotional impact on a women’s psychology, mental health, and wellbeing, including feelings of sadness, grief, anger, guilt, and depression [25]. Also, it has been linked to higher levels of anxiety, despair, and distress [5]. The intensity and duration of these emotions can vary from woman to woman and can last for weeks, months, or even years [26] and may persist into subsequent pregnancies [27].

The results of this study highlight the importance of seeking support after a miscarriage, as well as the various strategies that can be effective in reducing negative emotions. It is evident from previous scholars that receiving social support during and beyond miscarriage minimized certain emotions, such as depression and anxiety levels experienced by women after a miscarriage [28]. It was also reported that women who have experienced a miscarriage rely on two main networks for support: formal (healthcare providers) and informal (friends, family, and work colleagues). The formal care network was seen as the most trusted source of information while the informal network was the main source of tangible support [29]. The latter represented the larger percentage of reported network of support by the participants in our study. Having support can make a significant impact on reducing negative emotions and helping a person cope with the loss. Twenty-four valued aspects of care were found for women who have experienced a miscarriage. The most important aspect was being treated as an individual person rather than just a common condition. This was followed by providing understandable information about the cause of pregnancy loss, discussing patients’ distress, informing patients about pregnancy loss in the presence of a friend or partner, and making follow-up phone calls to support patients after a miscarriage [30]. In addition, it was reported that providing compassionate bereavement care and using appropriate terminology that acknowledge and respect the language preferences of parents to describe their loss as “a miscarriage” or “losing a baby” helped them cope with their loss [31]. Moreover, the proactive and compassionate care for women after a miscarriage, improved the overall experience and outcomes for the women who have suffered one [32]. Lately, online support groups for pregnancy loss have provided better support. They provide a confidential space to share experiences and emotions, as well as access to information and resources [25].

All studies have agreed that emotional support is very important, regardless of the uniqueness of everyone’s experience and healing journey. It is important to remember that people heal at their own pace and there is no right or wrong way to cope with a miscarriage. The healing process after a miscarriage is different for every woman and can take time, patience, and support.

Participants reported varied future pregnancy plans after miscarriage. Although there is no evidence of physiological benefit for delaying a pregnancy attempt after an early loss [33], a large percent of participants decided to postpone pregnancy. Their decision can be explained by the encouragement of gynecologists to wait a minimum of six months before the next conception to ensure an optimal obstetric and perinatal outcome [34], despite the need for further research on optimal timing for conception and its impact on obstetric and perinatal outcomes. Nearly 50% of our study participants initiated contraception immediately after the miscarriage, such as intrauterine devices and oral contraceptives. Almost equal percentages regarding contraception methods after miscarriage were reported in Roe et al. (2021), where 14% (33/97) selected a long-acting reversible method, 18% (44/97) a short-acting reversible method, and 8% (20/97) condoms or emergency contraception. Those who need contraception after the completion of miscarriage may safely initiate any method immediately [35].

On the contrary, women who desire another pregnancy need not delay conception but instead used traditional methods, including but not limited to fertility-enhancing herbs, cupping therapy, or medically assisted reproduction treatments. It was distressfully to discover that women who felt ashamed of miscarriage were three times more likely to get pregnant soon after the miscarriage. Women in Arabic countries, including Jordan, are held responsible for the health of their pregnancy [36] and may be blamed and accused upon miscarriage, particularly if the cause of the miscarriage is not certain [13, 37]. Such feelings can place a woman under intense scrutiny until she proves her ability in subsequent pregnancies. It is important to implement public health programs that increase women’s understanding of pregnancy and pregnancy loss, in order to alleviate any unwarranted feelings of shame that Jordanian women may be facing.

We acknowledge the limitations of our study, including the retrospective nature of our study and the use of multiple-choice questions, which introduce potential biases and limitations in understanding. The restriction on participant elaboration and the absence of an “other” category in the questionnaire further contributed to these limitations. Despite these constraints, our approach provided valuable insights into predefined response options and identified significant associations. Additionally, the sample composition, skewed towards highly educated women and recruited through social media, may limit the generalizability of our findings. Furthermore, our study design did not establish causality, highlighting the need for further research to explore underlying factors and mechanisms.

## Conclusion

Despite extensive research, the causes of many miscarriages remain unclear. The most common symptom of early pregnancy loss is vaginal bleeding, and surgical management is typically required. The emotional impact of a miscarriage on women is significant, influences women’s decisions to delay future pregnancy attempts, and underscores the need for adequate social support. Emotional support from healthcare providers, friends, and family is critical in helping women cope with their loss and move forward. Our study found that the lack of support was associated with increased negative feelings among women who experienced a miscarriage. It is important to provide couples and society with accurate information about pregnancy and miscarriage to avoid unnecessary blame and harm to affected women.

### Electronic supplementary material

Below is the link to the electronic supplementary material.

Supplementary Material 1

## Data Availability

All authors had full access to the data and materials. Data is available from the authors upon reasonable request.

## Data Availability

The datasets used and/or analyzed during the current study are available from the corresponding author upon reasonable request.
