# Supplementary material for: Navigating miscarriage in Jordan: understanding emotional responses and coping strategies
Source: BMC Pregnancy Childbirth. 2023 Oct 26;23:757. doi: 10.1186/s12884-023-06075-6 (PMC10601352; doi:10.1186/s12884-023-06075-6)
Supplement: Supplementary file 1 — Supplementary Material 1 [file 12884_2023_6075_MOESM1_ESM.docx]

**Navigating Miscarriage in Jordan: Understanding Emotional Responses and Coping Strategies**

Researchers from many Jordanian Universities are conducting a study on miscarriage among women in Jordan and we would like to know your perceptions, experiences, and practices after miscarriage. Please complete this 10-minute survey. Your responses are anonymous, your participation is voluntary and you are allowed to withdraw your participation at any time without giving excuses. Thank you for your participation.

**Do you agree to participate in the survey?**

- Yes
- No

| **Section One: Socio-demographic data** | | | | | |
| --- | --- | --- | --- | --- | --- |
| **Age**   - <22 years old - 22-34 years old - 35-44 years old - >44 years old | | | | | |
| **Place of residency**   - Irbid - Ajloun - Jerash - Mafraq - Balqa - Amman - Zarqa - Madaba - Karak - Tafilah - Ma’an - Aqaba | | | | | |
| **Employment**   - Employed - Unemployed | | | | | |
| **Educational level**   - Secondary School or lower - Diploma - Bache - Postgraduate | | | | | |
| **Monthly family income**   - <500 JD - 500-999 JD - 1000-1500 JD - >1500 JD | | | | | |
| **Number of children**   - None - 1 child - 2-3 children - 4 or more children | | | | | |
| **Number of miscarriages**   - Never - Once - Twice - Three time or more | | | | | |
| **Section Two: Women experience of miscarriage** | | | | | |
| **What was your miscarriage symptoms?** You may give more than one answer   - Vaginal bleeding - Abdominal pain - Passing tissue or clot - Fewer signs of pregnancy - No symptoms, suddenly discovered during regular checkup | | | | | |
| **How was your miscarriage managed or treated?** You may give more than one answer   - Observation, no medication - Medical treatment - Surgical treatment | | | | | |
| **What did the doctor tell you about the cause (Etiology) of your miscarriage?** You may give more than one answer   - Chromosomal abnormalities - Uterine factor - Thrombophilia & antiphospholipid syndrome - Endocrine abnormality - Infections - Unexplained | | | | | |
| **Section Three: Emotional responses and support** | | | | | |
| **Please rate the following statements on a scale from 1 to 5 with 1= strongly disagree and 5= strongly agree** | | | | | |
| **How did you feel after miscarriage?** | **Strongly Agree** | **Agree** | **Neutral** | **Disagree** | **Strongly Disagree** |
| I was extremely upset: like the loss of a child |  |  |  |  |  |
| I felt guilty |  |  |  |  |  |
| I felt alone |  |  |  |  |  |
| I felt ashamed |  |  |  |  |  |
| I was not upset: only an inconvenience |  |  |  |  |  |
| **What did you do after a miscarriage?** You may give more than one answer   - I avoided having sex for two weeks - I waited more than two months before trying to become pregnant again - I used intrauterine birth control method immediately - I used other birth control method immediately - I had spiritual treatment/ praying/ Quran recitation - I had cupping therapy - I used Vitex or Majorana plants - I did in vitro fertilization (IVF) - Nothing | | | | | |
| **What strategies did you use to avoid or manage bad emotions after miscarriages?** You may give more than one answer   - Receiving social support - Consulting psychologist and had psychological counselling - Consulting psychologist and took psychotropics (prescribed pharmacological agents) - Home relaxing herbal remedies such as peppermint, lavender, chamomile, ginger, rosemary - Over-the-counter relaxing medications - Stress relieving activities such as diet and exercise - Taking vitamins - Spiritual therapy - Nothing - Other, specify: _____ | | | | | |
| **In your opinion, which of the previous strategies improved your emotions?** You may give more than one answer   - Receiving social support - Consulting psychologist and had psychological counselling - Consulting psychologist and took psychotropics (prescribed pharmacological agents) - Home relaxing herbal remedies such as peppermint, lavender, chamomile, ginger, rosemary - Over-the-counter relaxing medications - Stress relieving activities such as diet and exercise - Taking vitamins - Spiritual therapy - None - Other | | | | | |
| **Who did mostly support you after miscarriage?** You may give more than one answer   - My family - My husband - Friends - Medical staff - Nobody | | | | | |
